# Associations of Type 2 Diabetes, Body Composition, and Insulin Resistance with Bone Parameters: The Dubbo Osteoporosis Epidemiology Study

**DOI:** 10.1002/jbm4.10780

**Published:** 2023-06-08

**Authors:** Angela Sheu, Robert D. Blank, Thach Tran, Dana Bliuc, Jerry R. Greenfield, Christopher P. White, Jacqueline R. Center

**Affiliations:** ^1^ Skeletal Diseases Program Garvan Institute of Medical Research Sydney NSW Australia; ^2^ School of Clinical Medicine, UNSW Medicine and Health, St Vincent's Clinical Campus, Faculty of Medicine and Health UNSW Sydney Sydney NSW Australia; ^3^ Department of Endocrinology and Diabetes St Vincent's Hospital Sydney NSW Australia; ^4^ School of Clinical Medicine, Prince of Wales Clinical Campus, Faculty of Medicine and Health UNSW Sydney Sydney NSW Australia; ^5^ Department of Endocrinology and Metabolism Prince of Wales Hospital Sydney NSW Australia

**Keywords:** BIOCHEMICAL MARKERS OF BONE TURNOVER, BONE‐FAT INTERACTIONS, DUAL‐ENERGY X‐RAY ABSORPTIOMETRY, FRACTURE RISK ASSESSMENT, STATISTICAL METHODS

## Abstract

Type 2 diabetes (T2D) may be associated with increased risk of fractures, despite preserved bone mineral density (BMD). Obesity and insulin resistance (IR) may have separate effects on bone turnover and bone strength, which contribute to skeletal fragility. We characterized and assessed the relative associations of obesity, body composition, IR, and T2D on bone turnover markers (BTMs), BMD, and advanced hip analysis (AHA). In this cross‐sectional analysis of Dubbo Osteoporosis Epidemiology Study, 525 (61.3% women) participants were grouped according to T2D, IR (homeostasis model assessment insulin resistance [HOMA‐IR] </≥2.5), and BMI (</≥25 kg/m^2^): insulin‐sensitive lean (IS‐L), insulin‐sensitive overweight/obese (IS‐O), insulin‐resistant (IR), and T2D. BMD, AHA, and body composition, including visceral adipose tissue (VAT) (on dual‐energy x‐ray absorptiometry scan) and fasting BTMs, were assessed. Analyses performed using Bayesian model averaging and principal component analysis. T2D was associated with low BTMs (by 26%–30% [95% confidence interval [CI] 11%–46%] in women, 35% [95% CI 18%–48%] in men compared to IS‐L), which persisted after adjustment for VAT. BTMs were similar among IR/IS‐O/IS‐L. BMD was similar among T2D/IR/IS‐O; BMD was low only in IS‐L. All groups were similar after adjustment for BMI. Similarly, AHA components were lowest in IS‐L (attenuated following adjustment). On multivariate analysis, T2D was independently associated with BTMs. IR was also associated with C‐terminal telopeptide of type 1 collagen in men. Age and body size were the strongest independent contributors to BMD and AHA. VAT was inversely associated with section modulus, cross‐sectional area, cross‐sectional moment of inertia in women, and hip axis length in men. Low bone turnover is associated with T2D and IR (in men), while BMD and hip strength/geometry are predominantly associated with body size. VAT, indicative of dysglycemia, is also associated with impaired bone geometry. Establishing the role of BTMs and AHA fracture risk may improve skeletal assessment in T2D people. © 2023 The Authors. *JBMR Plus* published by Wiley Periodicals LLC on behalf of American Society for Bone and Mineral Research.

## Graphical abstract

Associations between metabolic states and bone parameters:

- Low bone turnover in type 2 diabetes and insulin resistance.
- BMD and hip strength/geometry positively associated with body size but negatively with visceral adiposity.

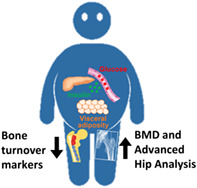

## Introduction

Type 2 diabetes (T2D) is associated with increased risk of fractures, particularly at the hip, despite preserved bone mineral density (BMD).^(^ ^1^, ^2^ ^)^ BMD (usually measured by dual‐energy x‐ray absorptiometry [DXA]) contributes to fracture risk as the main determinant of bone strength, but it does not capture changes in microarchitecture, bone turnover, and bone strength. T2D is associated with several changes in bone measurements, including low bone turnover and inferior microarchitecture (reviewed in Sheu et al.^(^ ^3^ ^)^). The hallmark of diabetic osteopathy appears to be maladaptive skeletal loading with reduced capacity under loading despite preserved BMD, partly due to impaired cortical parameters. Alternative imaging modalities, such as quantitative CT and trabecular bone score, can assess microarchitectural changes in T2D; however, their use in clinical practice are limited, thereby preventing widespread assessment of T2D patients. Advanced hip analysis (AHA) uses measurements from a hip DXA scan to estimate bone strength and microarchitecture.^(^ ^4^ ^)^ In the general population, some of these indices predict fracture risk independently of BMD.^(^ ^5^, ^6^, ^7^, ^8^ ^)^ Although the resolution of these scans is limited, the convenience of acquiring multiple structural and strength properties from a single scan may improve the assessment of bone fragility in people with T2D.

However, previous studies examining AHA in T2D are conflicting. Women with T2D may have elevated/better,^(^ ^9^ ^)^ similar,^(^ ^10^, ^11^ ^)^ or lower/worse^(^ ^12^, ^13^ ^)^ AHA parameters. Some studies only found differences when adjusted for body size or lean body mass.^(^ ^10^, ^12^, ^13^ ^)^ In those with and without T2D, more severe dysglycemia (e.g., presence of prediabetes, higher insulin resistance [IR], or HbA1c) may be associated with more significant changes.^(^ ^9^, ^11^, ^14^ ^)^ There may also be sex differences, although few studies have included male subjects.^(^ ^10^, ^13^, ^14^, ^15^, ^16^ ^)^ The relationship between AHA across a spectrum of female and male subjects with obesity, IR, and T2D has not been evaluated.

Although obesity is a risk factor for T2D, not all obese people have metabolic sequelae; rather, adverse metabolic effects (e.g., IR, dyslipidemia) are driven by visceral adipose tissue (VAT), located deep within the abdomen (reviewed in Samocha‐Bonet et al.^(^ ^17^ ^)^). VAT and IR may also affect bone, independently of the mechanical and hormonal effects of obesity and subcutaneous fat. We previously showed inducible suppression of bone turnover markers (BTMs) in insulin‐sensitive subjects with supraphysiological levels of insulin, suggesting that hyperinsulinemia drives bone turnover suppression in T2D.^(^ ^18^ ^)^ However, more severe hyperglycemia has not been consistently associated with lower BTMs^(^ ^19^, ^20^ ^)^ or higher fracture risk.^(^ ^21^, ^22^ ^)^ It is therefore unclear how obesity, IR, and VAT independently and cumulatively relate to bone metabolism and fracture risk within T2D individuals.

We hypothesized that T2D was associated with worse hip strength and geometry indices on AHA and that IR and VAT were independently associated with BTM, BMD, and AHA parameters. The aim of this study was to dissect the relationship between bone parameters (BTMs, BMD, and AHA) with metabolic parameters (body composition, VAT, and IR) in lean and overweight subjects, with and without IR and T2D, to determine which metabolic phenotype was most strongly associated with a fracture‐promoting bone phenotype.

## Methods

### Study design and participants

This was a cross‐sectional study from the Dubbo Osteoporosis Epidemiology Study (DOES), an ongoing longitudinal population‐based study of people aged ≥60 years living in Dubbo, New South Wales, Australia.^(^ ^23^, ^24^ ^)^ Every 2–3 years, participants were assessed for medical and lifestyle factors and had a DXA scan performed. From 2000, fasting morning serum was collected and stored at each visit for all participants. As there were some differences between the participants recruited before and after 2000,^(^ ^25^ ^)^ this cohort only included participants recruited from the year 2000 onward (DOES2). Participants were included in this analysis if they had a concurrent fasting serum sample and DXA scan available for analysis of all measurements (BMD, body composition, and VAT). Subjects were excluded if they had bone diseases other than osteoporosis (such as Paget's disease, hyperparathyroidism) or malignancy, type 1 diabetes, or advanced renal disease (self‐diagnosis or calculated estimated glomerular filtration rate [eGFR] <30 mL/min/1.73 m^2^).

All participants provided written informed consent. The study was approved by St Vincent's Hospital Human Research Ethics Committee (NEAF AU/1/80C517).

### Subject assignment to groups

The participants (*n* = 525) were divided into four groups according to T2D, IR (homeostasis model assessment insulin resistance [HOMA‐IR] </≥2.5),^(^ ^26^, ^27^ ^)^ then BMI (</≥25 kg/m^2^): insulin‐sensitive lean (IS‐L; BMI <25 kg/m^2^ and HOMA‐IR <2.5, *n* = 131), insulin‐sensitive overweight/obese (IS‐O; BMI ≥25 kg/m^2^ and HOMA‐IR <2.5, *n* = 193), insulin‐resistant (IR, HOMA‐IR ≥2.5, *n* = 132), and T2D (*n* = 69).

T2D was defined as either self‐reported diagnosis, taking anti‐hyperglycaemic medications, or fasting serum glucose ≥7.0 mmol/L. As HOMA‐IR is an unreliable measurement of IR in T2D, HOMA‐IR was not calculated in these subjects.^(^ ^27^ ^)^

### BMD and AHA

BMD and AHA were measured by DXA using a GE Lunar Prodigy Pro Densitometer (Madison, WI, USA). BMD was recorded at the femoral neck (FN), total hip (TH), and lumbar spine (LS) (coefficient of variation (CV) 1%–3%, 1%–3%, 1%–5%, respectively, for normal subjects).

**Fig. 1:**
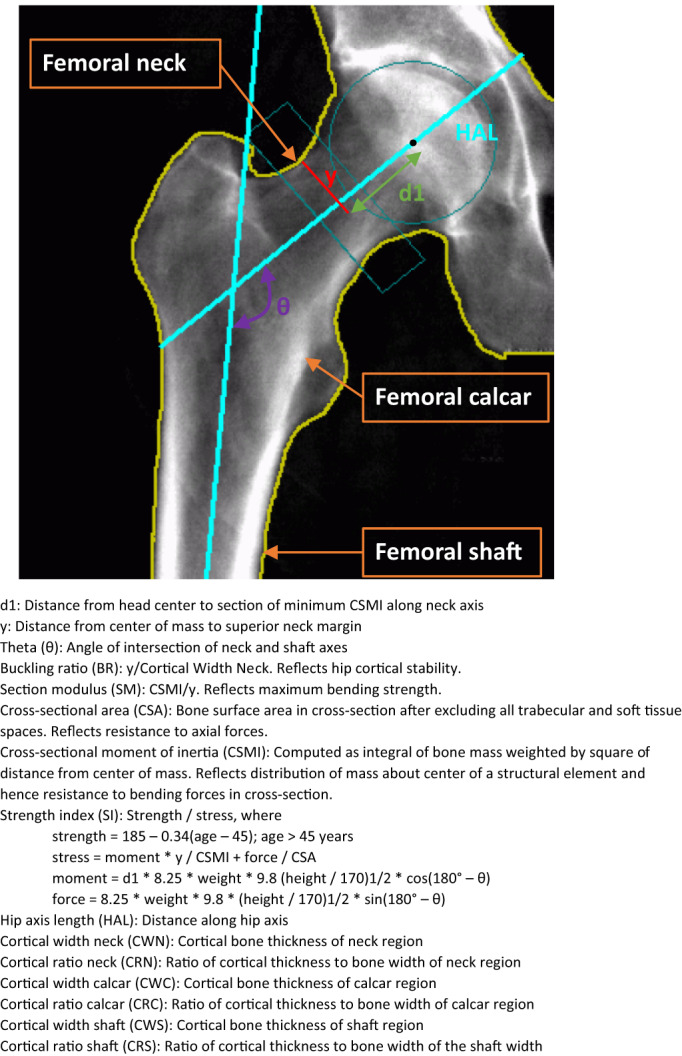
Components measured in advanced hip analysis (AHA).

There were 456/525 scans available for AHA analysis. AHA parameters were measured using the GE Lunar DXA hip image retrieved from enCORE software version 17.^(^ ^4^ ^)^ There are five calculated measures of strength at the FN (Fig. 1): buckling ratio (BR), a measurement of hip cortical stability where a higher value confers worse resistance to compressive force; section modulus (SM), maximum bending strength; cross‐sectional area (CSA), reflecting resistance to axial forces; cross‐sectional moment of inertia (CSMI), resistance to bending forces; and strength index (SI), a composite score that takes into account age, height, and weight. The hip axis length (HAL) is the distance measured from the base of the greater trochanter to the inner pelvic rim.

Structural hip geometry measurements include cortical bone thickness and the ratio of the cortical thickness to bone width measured at the femoral neck, calcar, and shaft.

### Body composition and visceral adiposity

Body composition (total and regional body measures of fat and lean mass) was measured on a total body scan performed at the same time as BMD (DXA). Images were analyzed according to manufacturer's instructions. The CVs for fat mass and lean mass measurements are 2.9% and 1.5%, respectively. VAT mass (kg) was calculated from the android region on the DXA scan using the enCORE CoreScan software. An additional central abdominal compartment, 10 pixels high from the pelvic brim and as wide as the lateral points of the rib cage, was specified to delineate between VAT and non‐VAT central adiposity.

### Biochemical measurements

Blood samples were collected from participants between 8 and 10 am following an overnight fast of 10–12 h. Serum was stored at −80°C and all samples were analyzed together.

Serum insulin, osteocalcin (OC), procollagen type 1 N‐terminal propeptide (P1NP), and collagen type 1 crosslinked C‐terminal telopeptide (CTX) were analyzed using automated electroluminescent immunoassays on the Roche Cobas e100. The respective interassay CVs are 4.9%, 2%, 3.7%, and 4.1%. Glucose and creatinine were analyzed by enzymatic method on the Roche Cobas c701. Interassay CVs were 1.0% and 1.4%. 25‐hydroxyvitamin D (25OHD) was analyzed by liquid chromatography‐mass spectrometry by AB Sciex 4000 QTrap. Renal function was determined by calculating the eGFR by the CKD‐EPI 2021 equation.^(^ ^28^ ^)^

### Statistical analyses

Data not normally distributed were log‐transformed for analysis. All analyses were sex‐specific. Baseline variables, metabolic measurements (body composition and VAT), and the main outcome measurements (BTMs, BMD, and AHA) were compared between the four groups (IS‐L, IS‐O, IR, T2D) using chi‐squared tests for categorical variables and one‐way analysis of variance (ANOVA) with post‐hoc Bonferroni testing for multiple comparisons, for continuous variables. Measured parameters were also compared after adjusting for BMI, and if significant, total body fat (TBF), lean body mass (LBM), and VAT. AHA measurements were also adjusted for FNBMD. Correlation analyses using the Pearson correlation measure were performed between bone and metabolic parameters.

Two statistical approaches were used to determine the associations of the metabolic components and the bone parameters whilst considering the significant collinearity of the many variables. Bayesian model averaging (BMA) identified variables based on the most parsimonious model with the greatest discriminatory power^(^ ^29^ ^)^ for each of the outcomes of interest. Regression analyses was performed to estimate the effect size of each selected variable. The 18 outcomes of interest were the BTMs (three markers), BMD (three sites) and AHA (12 components). Covariates that were entered into the BMA for consideration included the four groups, metabolic components (height, weight, body composition, VAT), age, biochemistry (serum 25OHD and eGFR), and lifestyle factors (smoking status, alcohol excess [defined as >10 or >14 standard drinks per week for women and men, respectively]). The analyses were repeated excluding T2D subjects and “group,” with glucose, insulin, and HOMA‐IR levels as additional candidate variables.

Given that many of the variables are collinear, principal component analysis (PCA) was performed as a secondary exploratory analysis to corroborate the findings from the BMA.^(^ ^30^ ^)^ Principal components (PCs) were created to reduce the variables into the fewest number of independent “categories.” The 18 bone outcomes (listed earlier for the BMA) were standardized to the mean and included in the PCA. All of the covariates mentioned earlier for the BMA, apart from “group,” were also standardized to the mean and included. “Group” was excluded as it contained four levels and could not be converted into a linear form for PCA. Analyses were repeated without T2D subjects in the same way as for BMA. Varimax rotation was used to maximize the saturation of each PC. PCs were included if their eigenvalues were >1.0. Associations between the PCs and the bone parameters were determined by multivariable regression analysis.

Two‐sided *p* values <0.05 were regarded as significant. Statistical analyses were performed using SAS, version 9 (SAS Institute, Inc., Cary, NC, USA) and the BMA package in R (R Foundation, Vienna, Austria; https://www.rproject.org).

## Results

### Baseline characteristics

In total, there were 525 participants with available data for analysis. Clinical and biochemical characteristics of the four groups are shown in Table 1. The groups did not differ in age. BMI was not significantly different between the IS‐O, IR, and T2D groups. Prior fracture and previous osteoporosis treatment were not different between groups and were higher in women than men. The median duration of T2D was 5.4 (3.5–10.7) and 5.5 (1.8–8.1) years in women and men, respectively. The majority of T2D subjects were managed with oral agents; 4/34 (12%) women and 3/35 (9%) men were on insulin treatment. As expected, there were differences in fasting glucose and insulin levels among the four groups.

**Table 1:** Baseline characteristics of study participants

Characteristic	Women (*n* = 322)					Men (*n* = 203)				
	IS‐L (*n* = 101)	IS‐O (*n* = 104)	IR (*n* = 83)	T2D (*n* = 34)	*p* value	IS‐L (*n* = 30)	IS‐O (*n* = 89)	IR (*n* = 49)	T2D (*n* = 35)	*p* value
Age (years)	68.0 ± 4.7	67.7 ± 4.7	68.1 ± 4.5	68.7 ± 4.2	0.71	68.8 ± 4.4	68.8 ± 4.3	69.0 ± 4.3	70.8 ± 5.8	0.19
Height (cm)	161.5 ± 6.6	160.3 ± 5.6	159.8 ± 5.5	158.6 ± 5.5	0.06	173.3 ± 5.1	173.8 ± 6.6	172.5 ± 6.0	170.7 ± 6.7	0.10
Weight (kg)	60.0 ± 6.7	73.4 ± 7.0	74.4 ± 11.6	75.5 ± 12.0	NA	68.9 ± 6.0	84.1 ± 9.9	84.3 ± 10.4	88.4 ± 13.4	NA
BMI (kg/m^2^)	23.0 ± 1.6	28.6 ± 2.6	29.1 ± 4.1	30.0 ± 4.1	NA	22.9 ± 1.5	27.8 ± 2.6	28.3 ± 2.8	30.2 ± 3.6	NA
Smoker	7 (7%)	7 (7%)	3 (4%)	4 (12%)	0.44	3 (10%)	5 (6%)	5 (10%)	4 (11%)	0.65
Alcohol	14 (14%)	12 (12%)	12 (14%)	1 (3%)	0.33	10 (33%)	25 (28%)	8 (16%)	2 (6%)	0.02
Falls	34 (34%)	39 (38%)	35 (42%)	18 (53%)	0.23	11 (37%)	30 (34%)	15 (31%)	7 (21%)	0.48
Osteoporosis treatment	18 (18%)	18 (17%)	12 (14%)	6 (18%)	0.93	2 (7%)	3 (3%)	3 (6%)	1 (3%)	0.77
Prior fracture	20 (20%)	20 (19%)	18 (22%)	6 (18%)	0.96	7 (23%)	11 (12%)	5 (10%)	2 (6%)	0.17
T2D duration (years)	NA	NA	NA	5.4 (3.5–10.7)	NA	NA	NA	NA	5.5 (1.8–8.1)	NA
T2D treatment										
Diet				7 (21%)					11 (31%)	
Oral agents				23 (68%)					21 (60%)	
Insulin (+/− oral)	NA	NA	NA	4 (12%)	NA	NA	NA	NA	3 (9%)	NA
Biochemical										
Glucose (mmol/L)	4.8 (4.4–5.0)	4.7 (4.5–5.1)	**5.3 (4.9–5.6)** ^**b**^	**6.5 (5.3–7.2)** ^**b**^ ^,^ ^**c**^ ^,^ ^**d**^	<0.0001	4.8 (4.7–5.4)	5.1 (4.8–5.4)	5.2 (4.9–5.6)	**6.7 (5.3–7.6)** ^**a**^ ^,^ ^**b**^ ^,^ ^**c**^	<0.0001
Insulin (mU/L)	5.44 (7.47–4.54)	**7.55 (6.00–9.29)** ^**a**^	**15.23 (13.02–21.35)** ^**a**^ ^,^ ^**b**^	**18.65 (13.31–42.76)** ^**a**^ ^,^ ^**b**^ ^,^ ^**c**^	<0.0001	**5.97 (4.17–7.74)**	**7.69 (5.77–9.13)** ^**d**^	**15.49 (12.56–19.12)** ^**a**^ ^,^ ^**b**^	**16.34 (12.16–24.58)** ^**a**^ ^,^ ^**b**^	<0.0001
HOMA‐IR	1.13 (0.90–1.55)	1.61 (1.24–2.00)	3.62 (2.99–4.96)	NA	NA	1.28 (0.91–1.89)	1.73 (1.31–2.11)	3.73 (2.90–4.78)	NA	NA
25OHD (nmol/L)	72.7 ± 22.3	65.8 ± 19.8	71.8 ± 25.0	64.3 ± 20.3	0.06	78.4 ± 20.1	77.5 ± 21.7	68.1 ± 17.1	69.8 ± 22.3	**0.03**
Creatinine (μmol/L)	69.9 ± 13.5	68.8 ± 10.5	74.2 ± 17.4	72.4 ± 13.5	**0.048**	85.2 ± 14.2	92.0 ± 16.3	88.0 ± 17.6	88.1 ± 19.2	0.21
eGFR	82.1 ± 14.3	83.3 ± 12.2	77.9 ± 16.8	81.1 ± 14.3	0.07	84.6 ± 12.5	79.3 ± 13.7	82.9 ± 15.1	83.5 ± 14.6	0.2

*Note*: Values are shown as means ± SD, median (IQR), or number (%). *p* value for ANOVA (with Bonferroni correction) and chi‐squared test. Bolded values indicate *p* < 0.05.

Abbreviations: 25OHD, 25‐hydroxyvitamin D; CTX, C‐terminal telopeptide of type 1 collagen; eGFR, estimated glomerular filtration rate; HOMA‐IR, homeostasis model assessment insulin resistance; IR, insulin resistant; IS‐L, insulin sensitive lean; IS‐O insulin sensitive overweight; NA, not applicable; NS, not significant; OC, osteocalcin; P1NP, procollagen type 1 N propeptide; T2D, type 2 diabetes.

^a^ *p* < 0.01 versus IS‐L.

^b^ *p* < 0.01 versus IS‐O.

^c^ *p* < 0.01 versus IR.

^d^ *p* < 0.05 versus IS‐L.

^e^ *p* < 0.05 versus IS‐O.

### Bone and body composition parameters

Bone and body composition parameters are shown in Table 2.

**Table 2:** Measured metabolic and bone parameters of women (A) and men (B)

A. Women					
Characteristic	IS‐L	IS‐O	IR	T2D	Unadjusted *p* value (ANOVA)
	*N* = 101	*N* = 104	*N* = 83	*N* = 34	
Bone turnover markers					
CTX (μg/mL)	318 (211–441)	251 (189–399)	285 (166–416)	**217 (127‐311)** ^**a**,**e**,**f**^	0.03
OC (ng/mL)	22.6 (17.7–28.4)	19.3 (15.2–25.1)	20.5 (15.4–24.4)	**13.4 (10.3–22.0)** ^**a**,**b**,**c**^	<0.0001
P1NP (μg/L)	44.2 (32.1–55.9)	38.2 (27.8–52.8)	43.9 (30.4–52.5)	**31.7 (21.6–42.6)** ^**c**,**d**,**e**^	0.004
Bone mineral density					
FNBMD (g/cm^2^)	0.82 ± 0.11	0.88 ± 0.11	0.89 ± 0.14	0.89 ± 0.16	0.002
THBMD (g/cm^2^)	0.85 ± 0.12	0.94 ± 0.11	0.95 ± 0.15	0.97 ± 0.18	<0.0001
LSBMD (g/cm^2^)	1.03 ± 0.19	1.13 ± 0.19	1.14 ± 0.23	1.10 ± 0.19	0.0005
Body composition					
Total body fat (kg)	21.3 ± 5.1	31.2 ± 5.2	31.8 ± 8.3	32.1 ± 8.4	<0.0001
Lean body mass (kg)	36.0 ± 3.4	38.8 ± 3.1	39.0 ± 4.7	40.6 ± 5.2	<0.0001
Trunk fat mass (kg)	10.5 ± 2.7	15.9 ± 2.8	**17.0 ± 4.3** ^**d**,**e**^	**17.9 ± 5.1** ^**d**^	<0.0001
Trunk lean mass (kg)	18.3 ± 1.8	19.5 ± 2.3	19.9 ± 2.6	**21.4 ± 3.3** ^**b**,**c**^	<0.0001
Central fat mass (kg)	1.3 ± 0.4	1.9 ± 0.4	**2.2 ± 0.5** ^**a**,**b**^	**2.3 ± 0.7** ^**a**,**b**^	<0.0001
Central lean mass (kg)	2.7 (2.5–2.9)	2.8 (2.6–3.0)	2.8 (2.6–3.1)	3.0 (2.7–3.3)	0.0012
VAT mass (kg)	0.46 (0.30–0.59)	0.88 (0.63–1.15)	**1.10 (0.71–1.48)** ^**a**,**b**^	**1.30 (1.06–1.53)** ^**a**,**b**^	<0.0001
Advanced hip analysis					
BR	3.6 (2.7–4.5)	3.3 (2.5–4.8)	3.3 (2.3–4.4)	3.5 (2.7–4.5)	0.64
SM (cm^3^)	0.543 ± 0.103	0.571 ± 0.105	0.564 ± 0.146	0.594 ± 0.142	0.17
CSA (cm^2^)	1.26 ± 0.19	1.37 ± 0.20	1.34 ± 0.25	1.38 ± 0.26	0.004
CSMI (cm^4^)	0.92 ± 0.22	0.99 ± 0.22	0.95 ± 0.28	0.98 ± 0.30	0.30
SI	1.62 ± 0.38	1.41 ± 0.31^g^	1.40 ± 0.41^g^	1.50 ± 0.36^g^	0.0002
HAL (cm)	10.5 ± 0.61	10.6 ± 0.52	10.5 ± 0.53	10.5 ± 0.59	0.90
Cortical width neck (mm)	4.4 (3.8–6.5)	4.9 (3.7–6.6)	5.1 (4.0–6.8)	4.8 (3.7–5.7)	0.64
Cortical ratio neck (%)	15.4 (12.4–20.4)	16.3 (11.8–22.0)	16.7 (12.8–22.6)	15.8 (12.0–19.6)	0.60
Cortical width calcar (mm)	3.2 (2.9–4.0)	*3.8 (3.6–4.5)* ^*a*,*i*^	*4.3 (3.7–4.6)* ^*g*^	*4.2 (3.7–5.0)* ^*g,j*^	<0.0001
Cortical ratio calcar (%)	6.3 (5.2–7.5)	7.2 (6.4–8.2)	***7.9 (7.1–8.7)*** ^***d***, ***e***, ***h***, ***j***^	***7.9 (6.9–9.3)*** ^***d***, ***e***, ***h***, ***j***^	<0.0001
Cortical width shaft (mm)	4.5 (3.9–5.1)	*5.1 (4.5–5.7)* ^*g*^	*4.9 (4.5–5.7)* ^*i*^	*5.1 (4.1–6.3)* ^*i*^	<0.0001
Cortical ratio shaft (%)	14.4 (12.0–16.7)	*16.3 (14.4–19.4)* ^*g*^	*16.2 (14.4–19.2)* ^*i*^	*17.0 (13.8–19.8)* ^*i*^	<0.0001

B. Men					
Characteristic	IS‐L	IS‐O	IR	T2D	Unadjusted *p* value (ANOVA)
	*N* = 30	*N* = 89	*N* = 49	*N* = 35	
Bone turnover markers					
CTX (μg/mL)	320 (221–477)	284 (220–364)	**261 (168‐330)** ^**d**^	**241 (146‐286)** ^**b**,**d**^	0.0006
OC (ng/mL)	16.9 (13.9–21.6)	17.3 (15.3–20.9)	16.4 (14.1–20.3)	**15.1 (11.2–18.3)** ^**e**^	0.03
P1NP (μg/L)	35.9 (27.6–49.1)	34.5 (29.6–42.8)	37.0 (28.4–44.7)	30.7 (25.8–39.9)	0.44
Bone mineral density					
FNBMD (g/cm^2^)	0.88 ± 0.12	0.96 ± 0.13	0.93 ± 0.14	0.96 ± 0.13	0.0006
THBMD (g/cm^2^)	0.96 ± 0.15	1.03 ± 0.14	1.04 ± 0.15	1.07 ± 0.13	0.02
LSBMD (g/cm^2^)	1.13 ± 0.19	1.28 ± 0.20	1.26 ± 0.23	1.35 ± 0.22	0.0006
Body composition					
Total body fat (kg)	15.4 ± 4.9	24.3 ± 6.1	26.2 ± 6.6	28.2 ± 6.9	<0.0001
Lean body mass (kg)	50.4 ± 5.2	56.4 ± 6.1	**54.5 ± 6.2** ^**e**^	**56.4 ± 8.3** ^**e**^	0.0002
Trunk fat mass (kg)	9.4 ± 3.4	15.0 ± 3.5	**16.9 ± 4.1** ^**a**,**b**^	18.1 ± 4.4	<0.0001
Trunk lean mass (kg)	24.8 ± 2.5	27.4 ± 3.3	26.7 ± 3.1	28.2 ± 4.4	0.0003
Central fat mass (kg)	1.3 ± 0.5	2.0 ± 0.5	**2.4 ± 0.5** ^**a**,**b**^	**2.4 ± 0.5** ^**d**^	<0.0001
Central lean mass (kg)	3.2 (2.9–3.6)	3.3 (3.2–3.6)	**3.2 (3.0–3.5)** ^**e**^	**3.3 (3.1–3.6)** ^**e**^	0.13
VAT mass (kg)	0.82 (0.53–1.40)	**1.68 (1.29–1.93)** ^**a**^	**2.06 (1.57–2.61)** ^**a**,**b**^	**2.20 (1.73–3.17)** ^**a**^	<0.0001
Advanced hip analysis					
BR	4.5 (3.1–6.2)	3.6 (2.4–4.6)	3.9 (2.8–5.3)	3.0 (2.5–4.2)	0.06
SM (cm^3^)	0.808 ± 0.198	0.889 ± 0.196	0.892 ± 0.190	0.886 ± 0.166	0.24
CSA (cm^2^)	1.54 ± 0.25	1.70 ± 0.27	1.72 ± 0.27	1.71 ± 0.26	0.03
CSMI (cm^4^)	1.57 ± 0.46	1.73 ± 0.46	1.77 ± 0.47	1.71 ± 0.40	0.34
SI	1.63 ± 0.36	1.51 ± 0.32	1.51 ± 0.33	1.54 ± 0.50	0.50
HAL (cm)	12.4 ± 0.71	12.3 ± 0.80	12.1 ± 0.65	12.1 ± 0.63	0.46
Cortical width neck (mm)	4.2 (3.2–6.4)	5.4 (4.1–7.9)	5.0 (3.8–7.4)	6.4 (4.6–7.5)	0.06
Cortical ratio neck (%)	12.1 (9.0–17.6)	15.5 (11.8–22.7)	13.9 (10.2–20.0)	18.4 (12.9–20.8)	0.07
Cortical width calcar (mm)	3.6 (2.9–4.4)	3.9 (3.1–4.6)	***4.4 (3.7–5.5)*** ^***d***, ***e***, ***i***, ***j***^	***4.6 (4.1–5.9)*** ^***d***, ***e***, ***i***, ***j***^	0.003
Cortical ratio calcar (%)	6.2 (4.9–7.4)	6.5 (5.2–7.5)	***7.2 (6.1–9.2)*** ^***e***, ***j***^	***7.8 (6.7–9.7)*** ^***e***, ***i***^	0.006
Cortical width shaft (mm)	4.5 (3.3–6.3)	5.1 (4.5–6.3)	5.2 (4.5–6.3)	5.7 (4.5–6.3)	0.21
Cortical ratio shaft (%)	13.9 (10.2–19.1)	16.3 (13.5–18.8)	16.1 (12.9–18.6)	16.7 (13.5–17.9)	0.37

*Note*: Values are shown as means ± SD, median (IQR), or number (%). Listed *p* value reflects between‐group differences of unadjusted measurements by ANOVA. All measurements were also compared after adjusting for BMI. Bolded values indicate significant between‐group differences when adjusted for BMI. ^a^ *p* < 0.01 versus IS‐L, ^b^ *p* < 0.01 versus IS‐O, ^c^ *p* < 0.01 versus IR, ^d^ *p* < 0.05 versus IS‐L, ^e^ *p* < 0.05 versus IS‐O, ^f^ *p* < 0.05 versus IR when adjusted for BMI. Advanced hip analysis measurements were also adjusted for FNBMD. Italicized values indicate significant between‐group differences when adjusted for FNBMD. ^g^ *p* < 0.01 versus IS‐L, ^h^ *p* < 0.01 versus IS‐O, ^i^ *p* < 0.05 versus IS‐L, ^j^ *p* < 0.05 versus IS‐O when adjusted for FNBMD.

Abbreviations: BMD, bone mineral density; BR, buckling ratio; CSA, cross‐sectional area; CSMI, cross‐sectional moment of inertia; CTX, C‐terminal telopeptide of type 1 collagen; FN, femoral neck; HAL, hip axis length; HOMA‐IR, homeostasis model assessment insulin resistance; IR, insulin resistant; IS‐L, insulin‐sensitive lean; IS‐O insulin‐sensitive overweight; LS, lumbar spine; NA, not applicable; OC, osteocalcin; P1NP, procollagen type 1 N propeptide; SI, strength index; SM, section modulus; T2D, type 2 diabetes; TH, total hip; VAT, visceral adipose tissue.

**Fig. 2:**
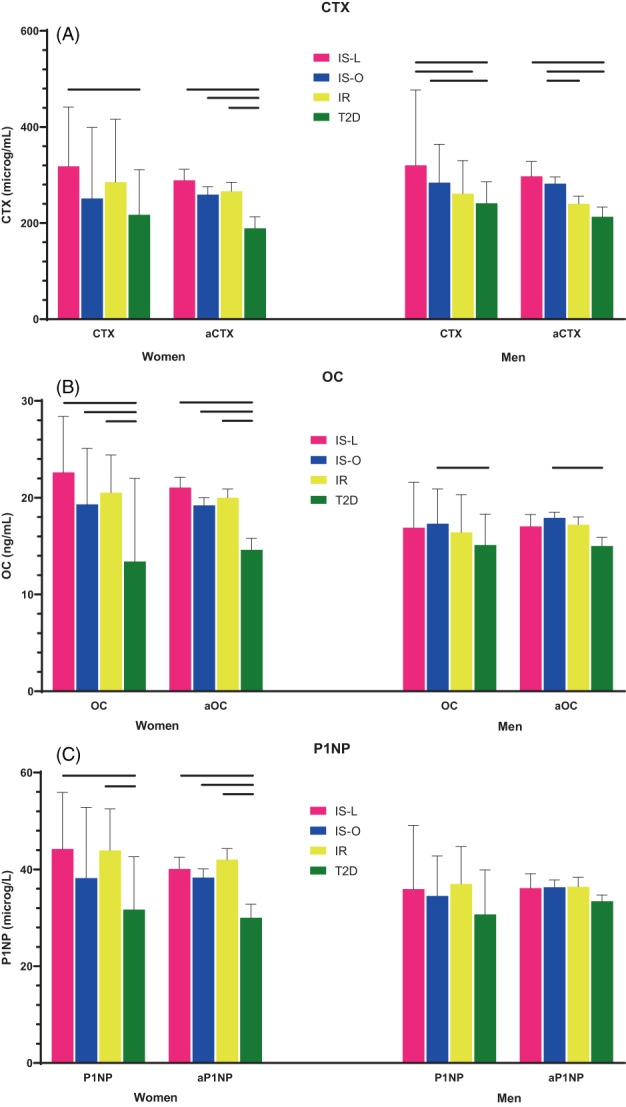
Bone turnover markers across the four groups, unadjusted and adjusted for BMI.

In women, all BTMs were lower only in T2D; BTMs did not differ among the other groups. BTMs remained lower in T2D when adjusted for BMI (Fig. 2). In men, only collagen type 1 crosslinked C‐terminal telopeptide (CTX) was significantly lower in T2D compared to the other groups. OC was lower in T2D compared to IS‐O, but not after adjustment for BMI. The same patterns were observed when adjustments were made for TBF, LBM, and VAT (data not shown).

**Fig. 3:**
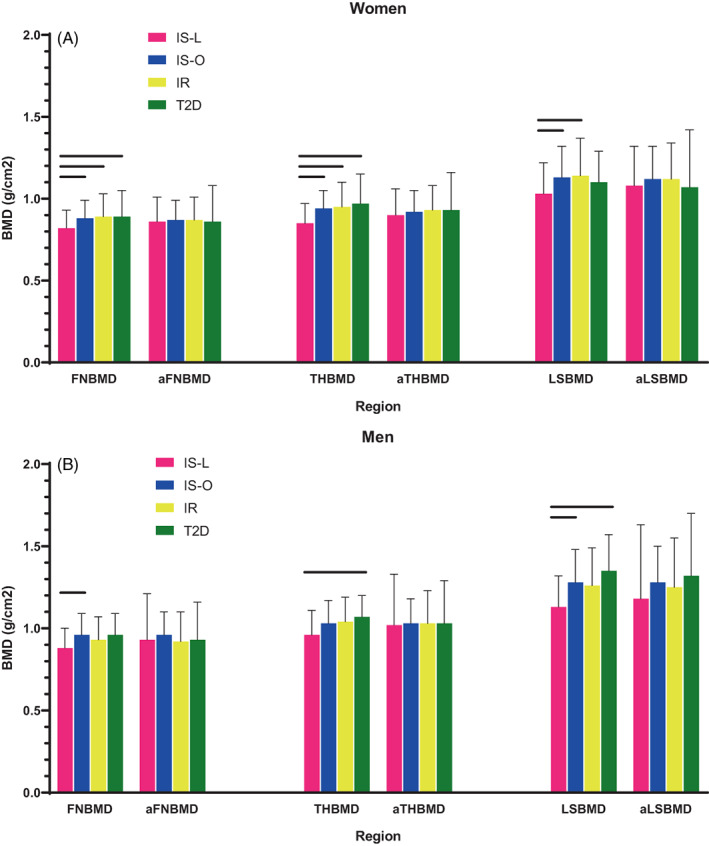
Bone mineral density across the four groups, unadjusted and adjusted for BMI, in women (*A*) and men (*B*).

BMD was lower only in IS‐L, but this difference was lost following adjustment for BMI (Fig. 3). There was no difference in unadjusted or adjusted BMD among T2D and IR/IS‐O.

For body composition, both fat and lean mass increased progressively from IS‐L to IS‐O, IR, and T2D. There was a trend to increasing VAT across the four groups.

The AHA parameters were similar across the four groups. CSA was lower in IS‐L, but this ceased to be significant following adjustment for BMI. SI was higher in IS‐L and remained significant when adjusted for FNBMD (in women) but not BMI. The calcar cortical width and ratio were significantly higher in IR/T2D, which persisted after either BMI or FNBMD adjustment. The shaft cortical width and ratio were lower in IS‐L women after FNBMD adjustment.

### Relationship between metabolic and bone parameters

Pearson correlation coefficients between the metabolic and bone parameters are shown in Table S1.

CTX and OC were negatively correlated with insulin level in women and VAT in men.

BMD was strongly correlated with BMI and most measures of body composition, including VAT. Hip BMD also correlated with insulin and HOMA‐IR in women.

Similarly, the AHA strength components were mostly correlated with BMI and body composition. In women, and less so in men, the AHA geometry components were also correlated with VAT, insulin, and HOMA‐IR.

### BMA multivariate regression

The final multivariate models, as chosen by BMA, for the whole cohort are shown in Table 3. T2D was significant for all BTMs in women and for CTX in men. When T2D subjects were excluded, HOMA‐IR was not significant (data not shown).

**Table 3:** Multivariable regression models to explain variability in BMD, BTM, and AHA according to metabolic parameters chosen by Bayesian model averaging (BMA) in women (A) and men (B).

A. Analyses in whole cohort of women				
Outcome	Adjusted *R* ^2^	Variable	Estimate (95% CI)^a^	*p* value
Bone turnover markers				
CTX	0.087	Age (/5 years)	**11.4% (3.5, 19.8)**	0.004
		Smoker	**−36.1% (−51.1, −16.4)**	0.001
		IS‐O	−7.6% (−21.6, 9.1)	0.35
		IR	−7.2% (−22.1, 10.6)	0.40
		T2D	**−30.9% (−46.2, −11.3)**	0.004
OC	0.127	Smoker	**−25.3% (−36.9, −11.6)**	0.0007
		eGFR	**−0.4% (−0.7, −0.1)**	0.02
		IS‐O	**−10.4% (−19.2, −0.7)**	0.04
		IR	−9.9% (−19.4, 0.6)	0.06
		T2D	**−31.9% (−41.8, −20.4)**	<0.0001
P1NP	0.074	Smoker	**−28.9% (−41.9, −12.9)**	0.001
		IS‐O	−7.1% (−17.9, 5.2)	0.24
		IR	0.6% (−11.8, 14.8)	0.93
		T2D	**−26.0% (−38.7, −10.8)**	0.002
Bone mineral density				
FNBMD (/SD)	0.182	Age (/5 years)	**−0.24 (−0.35, −0.13)**	<0.0001
		Weight (/5 kg)	**0.15 (0.11, 0.20)**	<0.0001
THBMD (/SD)	0.257	Age (/5 years)	**−0.25 (−0.35, −0.14)**	<0.0001
		Weight (/5 kg)	**0.21 (0.17, 0.26)**	<0.0001
		Height (/5 cm)	**−0.13 (−0.22, −0.05)**	0.003
LSBMD (/SD)	0.131	Weight (/5 kg)	**0.17 (0.12, 0.22)**	<0.0001
		Central lean mass	**−0.007% (−0.01, −0.003)**	0.0006
Advanced hip analysis				
BR		None		
SM	0.205	Height (/5 cm)	**0.02 (0.007, 0.03)**	0.001
		Weight (/5 kg)	**0.02 (0.02, 0.03)**	<0.0001
		VAT mass	**−0.0002% (−0.0003, −0.00005)**	0.004
CSA	0.240	Age (/5 years)	**−0.04 (−0.06, −0.01)**	0.004
		Weight (/5 kg)	**0.05 (0.04,0.07)**	<0.0001
		VAT mass	**−0.0003% (−0.0005, −0.0001)**	0.003
CSMI	0.238	Height (/5 cm)	**0.05 (0.03, 0.08)**	<0.0001
		Weight (/5 kg)	**0.05 (0.03, 0.06)**	<0.0001
		VAT mass	**−0.0003% (−0.0005, −0.00009)**	0.006
SI	0.080	Central fat mass	**−0.17 (−0.24, −0.10)**	<0.0001
HAL	0.328	Height (/5 cm)	**0.21 (0.16, 0.26)**	<0.0001
		Lean body mass	**0.03 (0.01, 0.04)**	0.0002
CWN		None		
CRN		None		
CWC	0.112	Trunk fat mass	**3.7% (2.0, 5.5)**	<0.0001
		Central fat mass	**−13.4% (−23.5, −1.9)**	0.02
CRC	0.112	Height (/5 cm)	**−4.2% (−6.9, −1.5)**	0.003
		Trunk fat mass	**3.8% (2.0, 5.6)**	<0.0001
		Central fat mass	**−14.5% (−24.8, −2.7)**	0.02
CWS	0.097	Weight (/5 kg)	**4.3% (2.7, 5.9)**	<0.0004
CRS	0.141	Age (/5 years)	**−6.6% (−10.0, −3.0)**	0.0005
		Height (/5 cm)	**−4.4% (−7.1, −1.6)**	0.003
		VAT mass	**0.1% (0.1, 0.2)**	<0.0001

B. Analyses in whole cohort of men				
Outcome	Adjusted *R* ^2^	Variable	Estimate (95% CI)^a^	*p* value
Bone turnover markers				
CTX	0.084	IS‐O	−11.1% (−26.5, 7.4)	0.22
		IR	**−24.8% (−38.9, −7.5)**	0.007
		T2D	**−34.7% (−48.1, −17.8)**	0.0003
OC	0.122	eGFR	**−0.5% (−0.8, −0.2)**	0.004
		Total body fat	**3.9% (1.1, 6.8)**	0.006
		Trunk fat mass	**−7.0% (−10.9, −3.0)**	0.001
P1NP		None		
Bone mineral density				
FNBMD (/SD)	0.281	Weight (/5 kg)	**1.98 (1.08, 2.88)**	<0.0001
		Total body fat	**−0.55 (−0.74, −0.35)**	<0.0001
		Lean body mass	**−0.38 (−0.56, −0.19)**	<0.0001
		Trunk fat mass	**0.33 (0.17, 0.49)**	<0.0001
		Central fat mass	**−0.56 (−1.04, −0.08)**	0.02
		IS‐O	0.21 (−0.21, 0.63)	0.33
		IR	−0.14 (−0.63, 0.34)	0.56
		T2D	−0.13 (−0.66, 0.40)	0.63
THBMD (/SD)	0.256	Weight (/5 kg)	**1.83 (0.95, 2.72)**	<0.0001
		Height (/5 cm)	**−0.15 (−0.27, −0.02)**	0.02
		Total body fat	**−0.49 (−0.68, −0.30)**	<0.0001
		Lean body mass	**−0.33 (−0.51, −0.15)**	0.0003
		Trunk fat mass	**0.28 (0.13, 0.43)**	0.0002
		Central fat mass	**−0.63 (−1.09, −0.16)**	0.008
LSBMD (/SD)	0.229	Age (/5 years)	**0.18 (0.04, 0.32)**	0.01
		Weight (/5 kg)	**1.75 (0.87, 2.64)**	0.0001
		Total body fat	**−0.44 (−0.63, −0.24)**	<0.0001
		Lean body mass	**−0.24 (−0.42, −0.06)**	0.01
		Trunk fat mass	**0.17 (0.05, 0.89)**	0.007
		Trunk lean mass	**−0.18 (−0.30, −0.07)**	0.001
Advanced hip analysis				
BR	0.070	Height (/5 cm)	**9.5% (4.1, 15.0)**	0.0005
SM	0.304	Weight (/5 kg)	**0.09 (0.06, 0.11)**	<0.0001
		Total body fat	**−0.02 (−0.02, −0.009)**	<0.0001
		Central lean mass	**−0.001% (−0.002, −0.0002)**	0.02
CSA	0.329	Weight (/5 kg)	**0.47 (0.22, 0.72)**	0.0003
		Total body fat	**−0.11 (−0.17, −0.06)**	<0.0001
		Lean body mass	**−0.08 (−0.13, −0.02)**	0.004
		Trunk fat mass	0.03 (−0.006, 0.06)	0.10
		Central lean mass	−0.0009% (−0.002, 0.0006)	0.24
CSMI	0.323	Height (/5 cm)	**0.13 (0.08, 0.19)**	<0.0001
		Weight (/5 kg)	**0.06 (0.03, 0.09)**	<0.0001
SI		None		
HAL	0.448	Height (/5 cm)	**0.20 (0.11, 0.30)**	<0.0001
		Lean body mass	**0.04 (0.02, 0.06)**	<0.0001
		VAT mass	**−0.001% (−0.002, −0.0007)**	<0.0001
CWN	0.066	Height (/5 cm)	**−8.6% (−13.4, −3.4)**	0.002
		Weight (/5 kg)	**4.0% (0.9, 7.1)**	0.01
CRN	0.069	Height (/5 kg)	**−8.4% (−12.8, −3.8)**	0.0005
CWC	0.094	Total body fat	**−4.7% (−7.8, −1.5)**	0.005
		Trunk fat mass	**9.5% (4.0, 15.4)**	0.0007
CRC	0.080	Total body fat	**−4.8% (−7.9, −1.6)**	0.004
		Trunk fat mass	**9.2% (3.8, 15.0)**	0.0009
CWS	0.057	Alcohol	**−17.8% (−27.4, −6.8)**	0.002
CRS	0.039	Alcohol	**−15.6% (−26.1, −3.7)**	0.01

*Note*: ^a^For the variables that were log‐transformed, parameter estimates have been back‐transformed, to denote the percentage change in dependent variable with every 1% change in independent variable. Non‐log‐transformed variables in displayed units. Bolded values indicate *p* < 0.05.

For BMD in women, age and overall body size (weight and height) were significant predictors. In men, total and trunk fat and lean masses were additionally predictive above weight and height. When T2D subjects were excluded, HOMA‐IR was significant for FN and LSBMD in men only (data not shown).

For the AHA strength components, in addition to body size, VAT predicted SM, CSA, and CSMI in women and HAL in men. As with BMD, several specific compartment masses also contributed to SM and CSA in men. When T2D subjects were excluded, HOMA‐IR contributed to HAL in men only (data not shown).

For the AHA geometry measurements, fat mass was significant for calcar measurements in both men and women. VAT was significant for cortical shaft ratio in women.

### PCA

PCA transforms raw data into fewer independent “categories,” or PCs. The top five PCs together contributed 76.7% and 80.8% of the variance of the bone characteristics in women and men, respectively (Table 4). A heuristic interpretation of the PCs involves renaming each according to the measures that contributed most. We acknowledge that labeling each of the PCs is subjective and open to various interpretations. PC1 reflects body size (with height and multiple fat and lean masses, 37%–40% of variance), PC2 reflects lean mass (positive contribution from lean masses and negative contribution from fat masses, 12%–19% of variance) and PC3 reflects age (9%–10% of variance). In women, PC4 is a mixed component (7%), and PC5 reflects lifestyle (7%). In men, PC4 reflects alcohol consumption (7%), and PC5 reflects smoking (7%). When T2D were excluded, insulin and HOMA‐IR were grouped with the PC2 for women and PC3 in men (not shown).

**Table 4:** Principal component analysis to categorize contributors to bone outcomes in women (A) and men (B).

A. Analyses in whole cohort of women					
Variable	PC1	PC2	PC3	PC4	PC5
Percentage of variance	40.2	12.4	9.6	7.6	6.9
Cumulative percentage of variance explained	40.2	52.7	62.2	69.8	76.7
Eigenvalue	6.04	1.87	1.44	1.14	1.03
Descriptive interpretation	Body size	Lean mass	Age	Mixed	Lifestyle
Age	−0.006	−0.129	**0.682**	0.061	−0.101
Height	**0.392**	0.030	−0.117	0.051	−0.047
Weight	0.101	**0.400**	−0.165	**0.603**	−0.112
BMI	**0.363**	−0.163	−0.045	−0.240	0.005
Smoker	0.016	0.181	0.091	**−0.396**	**0.562**
Alcohol	0.014	0.159	−0.045	0.241	**0.434**
Total body fat	**0.364**	−0.153	−0.167	0.035	−0.043
Lean body mass	**0.306**	**0.413**	0.065	0.059	−0.042
Trunk fat mass	**0.379**	−0.192	−0.123	0.036	0.023
Trunk lean mass	0.291	**0.402**	0.148	−0.024	−0.026
Central fat mass	**0.348**	−0.273	−0.004	0.008	0.106
Central lean mass	0.161	**0.406**	0.291	**−0.329**	0.063
VAT mass	**0.306**	−0.265	0.137	0.057	0.111
25OHD	−0.066	−0.112	−0.050	**0.303**	**0.662**
eGFR	−0.064	0.151	**−0.554**	**−0.385**	−0.028

B. Analyses in whole cohort of men					
Variable	PC1	PC2	PC3	PC4	PC5
Percentage of variance	37.0	18.9	10.2	7.7	7.1
Cumulative percentage of variance explained	37.0	55.8	66.1	73.7	80.8
Eigenvalue	5.54	2.83	1.53	1.15	1.06
Descriptive interpretation	Body size	Lean mass	Age	Alcohol	Smoking
Age	−0.088	−0.164	**0.489**	−0.247	0.051
Height	**0.409**	0.129	0.043	−0.001	−0.074
Weight	0.127	**0.363**	0.029	0.140	**−0.439**
BMI	**0.388**	−0.061	0.029	−0.084	0.170
Smoker	0.019	0.111	−0.226	−0.296	**0.673**
Alcohol	−0.015	0.047	−0.171	**0.735**	0.288
Total body fat	**0.384**	−0.190	−0.029	0.006	−0.029
Lean body mass	0.274	**0.422**	0.114	−0.014	−0.080
Trunk fat mass	**0.385**	−0.223	−0.016	0.032	−0.043
Trunk lean mass	0.267	**0.422**	0.095	−0.012	0.005
Central fat mass	**0.314**	**−0.332**	0.018	0.080	0.092
Central lean mass	0.102	**0.399**	0.094	−0.137	**0.387**
VAT mass	**0.318**	**−0.302**	0.033	0.073	0.047
25OHD	−0.068	0.043	**0.448**	**0.500**	0.244
eGFR	0.034	0.058	**−0.665**	0.051	−0.069

*Note*: The top five components that were above the 1.0‐eigenvalue threshold, contributed almost 77% of the total variability of the bone characteristics. The top five components that were above the 1.0‐eigenvalue threshold contributed 80% of the total variability of the bone characteristics. The highest weights (>0.3) are bolded.

Univariate regression was used to quantify the association between each PC and the bone parameters (Table S2). Overall, the PCs selected were aligned with the variables chosen by BMA. PC1 (body size) was significant for most BTMs in both women and men. Additionally, PC3–5 (age, adiposity, and lifestyle) were also significant in women, in keeping with the BMA (smoking and T2D). BMD was explained by PC1 (body size) and PC3 (age), which was also consistent with the BMA (age, weight, and fat masses). Similarly, just as body size and fat masses were selected for both the AHA strength and geometry components by the BMA, PC1 (body size) and PC2 (lean mass) were most consistently selected by PCA, particularly in women.

## Discussion

In this cross‐sectional study examining the relationship between detailed metabolic and bone characteristics, the relative associations of obesity versus IR versus T2D in both women and men were assessed. BTM suppression occurs in T2D women only, and in men with T2D and IR. Obesity and increased body size are associated with higher BMD and AHA parameters. Metabolically active VAT negatively associates with AHA measures that represent bending and axial strength in women and HAL in men. Two novel statistical approaches (BMA and PCA) to handle the collinearity of multiple parameters yielded consistent findings. Thus, VAT and T2D/IR are associated with altered bone structure and lower bone turnover, respectively, which may lead to impaired bone strength and quality and explain the increased propensity for fracture despite preserved BMD in some T2D people.

The elevated BMD in T2D is associated with concomitant obesity. Multivariable analysis suggested that total body size, rather than specific body compartments or metabolic parameters, contributed the greatest variability in BMD. Although most data in the general population suggest lean mass accounts for the greatest variance in BMD,^(^ ^31^, ^32^ ^)^ these studies were confounded due to the collinearity of lean and fat mass when measured by DXA and total body size. We used multiple statistical approaches to best understand the individual contributions of these collinear measurements, with our data supporting body size, rather than metabolic activity, as the predominant driver of BMD.

Our data also extend the evidence that T2D is associated with low bone turnover. In particular, there may be specific differences between bone resorption and formation, particularly between the sexes, and the relative contributions of IR. In women, low BTMs persisted in T2D only after adjusting for BMI and fat masses, and on multivariable analysis, all BTMs were 30% lower in T2D, with no other metabolic parameter selected for inclusion in the model. In men, CTX and OC (but not P1NP) were lower in T2D, which persisted after multiple adjustments. IR was also associated with lower CTX and was significant in the multivariable model. This is consistent with a study of 112 obese men (mean age 50 years, BMI 40 kg/m^2^), where both CTX and OC were low only in the T2D group (but not in obese subjects without T2D).^(^ ^33^ ^)^ We previously showed lower BTMs in obese IR and T2D individuals, with further suppression during supraphysiological insulin exposure in only insulin‐sensitive individuals, suggesting that IR drives bone turnover suppression.^(^ ^18^ ^)^ In contrast, in young healthy men (mean age 23 years, mean BMI 23 kg/m^2^), BTMs fell during hypoglycemic clamping but not following induction of hyperinsulinemia, suggesting effects from hypoglycemia (directly or indirectly) and not insulin.^(^ ^34^ ^)^ How chronic hyperglycemia, hyperinsulinemia, visceral adiposity, and sex interact to influence bone turnover within a T2D individual remains unclear. Fracture risk is generally associated with high BTMs in the general population,^(^ ^35^ ^)^ although some conditions with low bone turnover (e.g., acromegaly, adynamic bone disease in chronic kidney disease) are also associated with increased fractures. However, BTMs did not predict incident clinical fractures in older adults with T2D in one case–control study.^(^ ^36^ ^)^ Additional studies to understand the significance of low BTMs in T2D are warranted.

The existing data on the effect of T2D/IR on AHA are conflicting, and this study extends the association of dysglycemia, in addition to body weight, on AHA. In the Baltimore Longitudinal Study of Aging (BLSA), AHA parameters were inversely associated with both impaired glucose tolerance and T2D in women only after multivariate adjustment (including for BMI and FNBMD).^(^ ^10^ ^)^ In a smaller study of 134 non‐insulin‐requiring T2D subjects, VAT correlated with SM and CSA in women (but not men), though no further specific analyses on its associations were performed.^(^ ^16^ ^)^ Although we did not find significant differences in AHA parameters overall among the four groups, VAT mass was a significant independent associate of SM, CSA, and CSMI in women, independent of body weight, supporting the hypothesis that dysglycemia adversely affects hip structure and suggesting that we may have been underpowered to detect between‐group changes. It is possible that VAT preferentially affects different parts of the hip, resulting in changes in the AHA measures, though the specific mechanisms could not be determined in this cross‐sectional study.

In men, there was no relationship between the metabolic components (VAT, central fat mass, and HOMA‐IR) and AHA, raising the possibility that sex hormones may influence the relationship between dysglycemia and bone strength. In the PCA, adiposity appeared to contribute in women (especially in PC4), but this PC was notably absent in men. Overall, this is in keeping with the BLSA findings, where differences were only observed in women but not men.^(^ ^10^ ^)^ Similarly, the first study of AHA in T2D men (aged 30–79 years) found no association between AHA parameters and T2D.^(^ ^15^ ^)^ Given that VAT is a lower proportion of total body weight in T2D men compared to women, it is possible that the smaller effect of VAT is less discernible, especially in milder forms of T2D, or that sex‐related differences in bone remodeling or body composition may alter the bone‐metabolic interface. Further studies in both sexes are required to verify these differences and establish possible causes.

Fracture risk is elevated in T2D in some, though not all, studies; discrepancies may relate to the heterogeneity of T2D cohorts examined, given that T2D‐related characteristics mediate fracture risk.^(^ ^37^, ^38^, ^39^ ^)^ However, fracture risk in T2D is higher than a non‐T2D counterpart at a given BMD.^(^ ^40^ ^)^ The elevated fracture risk in T2D, despite normal/elevated BMD, appears to relate to changes in microarchitecture that may result in reduced strength load.^(^ ^3^ ^)^ Noninvasive assessment of hip strength is limited and not well validated. AHA can be calculated easily from hip DXA scans and may improve prediction of some fractures over BMD in the general population.^(^ ^5^, ^6^, ^7^, ^8^ ^)^ We hypothesized that T2D subjects would have worse AHA parameters, which may partially explain the increased fracture risk in T2D. We used two different statistical approaches to dissect the relative associations of the various metabolic components on multiple hip parameters. AHA measures correlated with BMI and body masses, and this was confirmed on both BMA and PCA, where overall body size and both lean and fat masses were significant. These data suggest that body weight may have a greater impact on hip geometry and therefore strength that outweighs the adverse effects from impaired metabolism. Although no causative conclusions can be drawn from this cross‐sectional study, we propose that decades of exposure to higher body weight have positive impacts on hip geometry and that the relatively recent negative effects of IR/dysglycemia may be insufficient to be detected on DXA, which is limited to macroscopic measurements.

T2D is associated with a unique fracture risk profile. Increased hip fractures have been most extensively studied, with less consistent evidence for nonhip fractures.^(^ ^38^ ^)^ Peripheral fractures, particularly at the wrist and lower leg (foot, ankle), also appear to be more common in T2D, though it is unclear if this risk is only in certain individuals with T2D (e.g., treated with insulin, comorbid diabetic microvascular complication ^(^ ^22^, ^41^, ^42^ ^)^). In our cohort of T2D subjects (characterized by short duration and few treated with insulin), we did not find an increased risk of fragility fractures,^(^ ^43^ ^)^ and therefore our subjects may not have progressed along the diabetic bone spectrum to result in increased fracture risk, despite beginning to develop some subtle bone changes. Insulin—whether endogenous or exogenous—appears to be a significant contributor to increased fracture risk, and since few of our subjects were treated with insulin, our population may not have had sufficient T2D osteopathy to have detectable between‐group differences. However, our data confirms that higher VAT is associated with poorer AHA parameters, and it is possible that bone strength at nonhip sites are similarly impaired by metabolic dysfunction. Studies investigating changes in bone structure and microarchitecture, and how these influence bone strength, at multiple sites and across a spectrum of well‐characterized T2D subjects would further our understanding of how T2D affects bone structure and the role of alternative imaging techniques in stratifying fracture risk.

In summary, low BTMs were associated with T2D in both sexes and IR in men, while BMD and AHA were associated with body size. No causative associations can be inferred, but this is consistent with an initial adaptive response of hip density/geometry to high body weight (over the preceding years and even decades), whereas low BTMs reflect the acute effects of T2D/IR on bone physiology. Postulated underlying mechanisms include accumulation of advanced glycation end products, which may impair cross‐linking of proteins and collagen leading to reduced stress load, and/or the presence of vascular complications, where diabetic microangiopathy may prevent bone angiogenesis and nutrient supply.^(^ ^38^ ^)^ These changes may impair bone turnover, potentially influencing fracture risk, before structural changes are observed.

The strengths of our study include detailed, simultaneously collected metabolic and bone phenotyping, including VAT measurements. We were able to separate subjects according to both weight and IR to allow quantification of their independent associations. Our study included both men and women, which added to the limited data available on men and highlighted potential differences between sexes. Two novel statistical approaches to handling collinearity yielded consistent findings.

We acknowledge the study's limitations. Because it is a cross‐sectional study, causation cannot be inferred, and only associations between metabolic and bone phenotypes can be drawn. Longitudinal studies would be helpful to assess the effect of metabolic changes on bone. Bone and body composition parameters were all measured by DXA and are at risk of collinearity. However, we used multiple statistical approaches to meaningfully identify independent associations. Although AHA measurements may partially explain fracture risk, they are unable to assess material properties of bone or provide any detailed microarchitecture assessment, both of which are likely to be affected in T2D. Additionally, despite the terminology used by the AHA, two‐dimensional DXA estimates of proximal femur breaking strength are not as well correlated with that derived by three‐dimensional finite‐element analyses using quantitative computed tomography data,^(^ ^44^ ^)^ and thus references to bone strength should be interpreted with caution. Our cohort included subjects with relatively mild T2D, so generalizability may be limited. Finally, although PCA reduces the number of collinear variables, the interpretation of the PCs is subjective and is only hypothesis generating. Nonetheless, the findings were consistent with the BMA analysis and provide additional confidence in the assessment of the multiple correlated components.

This study separated the associations of obesity versus IR and T2D with bone parameters, thereby strengthening the known relationships between metabolic and bone health. We showed that low BTMs were unique to T2D and IR, rather than obesity, and that VAT was independently adversely associated with hip structure, which could impact hip strength. Although changes are subtle on DXA, these findings confirm the need for alternative methods for assessing fracture risk in people with T2D. Further studies evaluating subjects with detailed T2D characteristics are warranted and may reveal therapeutic interventions that could reduce the burden of diabetic osteopathy.

## Author Contributions

**Angela Sheu:** Conceptualization; data curation; formal analysis; investigation; methodology; project administration; software; validation; writing – original draft; writing – review and editing. **Robert Blank:** Conceptualization; methodology; supervision; writing – review and editing. **Thach S Tran:** Investigation; methodology; resources; software; validation; writing – review and editing. **Dana Bliuc:** Investigation; methodology; resources; software; writing – review and editing. **Jerry R. Greenfield:** Conceptualization; funding acquisition; methodology; supervision; writing – review and editing. **Christopher White:** Conceptualization; funding acquisition; investigation; methodology; project administration; supervision; visualization; writing – review and editing. **Jacqueline R Center:** Conceptualization; funding acquisition; investigation; methodology; project administration; resources; software; supervision; writing – review and editing.

## Funding Information

This work was supported by the National Health Medical Research Council Australia Projects 1070187, 1008219, and 1073430. Other funding bodies were a St Vincent's Clinic Foundation grant, Osteoporosis Australia‐Amgen grant, and the Mrs Gibson and Ernst Heine Family Foundation. Angela Sheu is supported by NHMRC postgraduate, Diabetes Australia, and Osteoporosis Australia Royal Australian College of Physicians Research Entry scholarships. Dana Bliuc is supported by a fellowship from Australian and New Zealand Bone and Mineral Society. The authors are solely responsible for the contents of this paper, which do not reflect the views of NHMRC.

## Disclosures

Angela Sheu, Thach Tran, Dana Bliuc, Jerry R. Greenfield, and Christopher P. White have no competing interests to declare. Robert D. Blank reported receiving personal fees from Bristol‐Myers Squibb Company, stock ownership in Abbott Laboratories, AbbVie, Amgen Inc., GSK PLC, Johnson & Johnson, and Procter & Gamble Co., serving on the advisory board of Amgen, having ownership interest in JangoBio outside the submitted work, serving as treasurer of the International Federation of Musculoskeletal Research Societies and the International Society for Clinical Densitometry, receiving an editorial stipend from Elsevier, receiving royalties from Wolters Kluwer NV, and serving on the board of the Asia‐Pacific Fragility Fracture Alliance. Jacqueline R. Center has consulted for and/or given educational talks for Amgen, Actavis, and Bayer.

### Peer Review

The peer review history for this article is available at https://www.webofscience.com/api/gateway/wos/peer‐review/10.1002/jbm4.10780.

## Supporting information


**Table S1.** Pearson correlation coefficients (*r* ^*p* value^) between metabolic and bone parameters in women (A) and men (B).
**Table S2.** Linear regression models to explain variability in BMD, BTM, and AHA according to principal component analysis in women (A) and men (B).

## Data Availability

The data sets analyzed during the current study are not publicly available due to ethics restrictions but are available from the corresponding author on reasonable request.
