# Supplementary material for: Associations of Type 2 Diabetes, Body Composition, and Insulin Resistance with Bone Parameters: The Dubbo Osteoporosis Epidemiology Study
Source: JBMR Plus. 2023 Jun 8;7(9):e10780. doi: 10.1002/jbm4.10780 (PMC10494511; doi:10.1002/jbm4.10780)
Supplement: Supplementary file 1 — Table S1. Pearson correlation coefficients (r p value) between metabolic and bone parameters in women (A) and men (B). Table S2. Linear regression models to explain variability in BMD, BTM, and AHA according to principal component analysis in women (A) and men (B). [file JBM4-7-e10780-s001.docx]

## Supplementary Table 1. Pearson correlation coefficients (r^p-value^) between metabolic and bone parameters in women (A) and men (B).

|  | **BMI** | **Total Fat** | **Total Lean** | **Trunk Fat** | **Trunk Lean** | **Central Fat** | **Central Lean** | **VAT** | **Glucose** | **Insulin** | **HOMA-IR** |
| --- | --- | --- | --- | --- | --- | --- | --- | --- | --- | --- | --- |
| **BTM** |  |  |  |  |  |  |  |  |  |  |  |
| **CTX** | -0.06 | -0.04 | -0.06 | -0.04 | -0.10 | 0.01 | -0.04 | 0.04 | 0.04 | **-0.13^0.02^** | -0.04 |
| **OC** | **-0.15^0.008^** | -0.11 | **-0.12^0.03^** | **-0.13^0.02^** | **-0.18^0.001^** | -0.10 | **-0.15^0.009^** | -0.05 | -0.09 | **-0.11^0.048^** | -0.02 |
| **P1NP** | -0.08 | -0.08 | -0.08 | -0.09 | **-0.15^0.009^** | -0.08 | -0.11 | -0.07 | -0.09 | -0.02 | 0.05 |
| **BMD** |  |  |  |  |  |  |  |  |  |  |  |
| **LS** | **0.26^<0.0001^** | **0.30^<0.0001^** | **0.18^0.001^** | **0.33^<0.0001^** | **0.12^0.03^** | **0.24^<0.0001^** | -0.06 | **0.18^0.002^** | -0.01 | 0.05 | 0.08 |
| **FN** | **0.33^<0.0001^** | **0.34^<0.0001^** | **0.25^<0.0001^** | **0.33^<0.0001^** | **0.16^0.004^** | **0.22^<0.0001^** | 0.07 | 0.11 | 0.04 | **0.11^0.045^** | 0.10 |
| **TH** | **0.45^<0.0001^** | **0.42^<0.0001^** | **0.30^<0.0001^** | **0.44^<0.0001^** | **0.23^<0.0001^** | **0.33^<0.0001^** | 0.07 | **0.23^<0.0001^** | 0.08 | **0.19^0.0007^** | **0.15^0.01^** |
| **AHA** |  |  |  |  |  |  |  |  |  |  |  |
| **BR** | -0.05 | 0.01 | 0.03 | 0.00 | 0.05 | 0.01 | 0.03 | -0.01 | 0.05 | **-0.12^0.04^** | **-0.13^0.04^** |
| **SM** | **0.21^0.0003^** | **0.26^<0.0001^** | **0.38^<0.0001^** | **0.25^<0.0001^** | **0.31^<0.0001^** | **0.15^0.01^** | **0.18^0.002^** | 0.07 | 0.04 | 0.02 | 0.01 |
| **CSA** | **0.29^<0.0001^** | **0.34^<0.0001^** | **0.35^<0.0001^** | **0.33^<0.0001^** | **0.26^<0.0001^** | **0.21^0.0005^** | 0.12 | 0.10 | 0.02 | 0.09 | 0.05 |
| **CSMI** | **0.19^0.001^** | **0.27^<0.0001^** | **0.39^<0.0001^** | **0.25^<0.0001^** | **0.34^<0.0001^** | **0.15^0.01^** | **0.21^0.0004^** | 0.07 | 0.00 | -0.02 | -0.04 |
| **SI** | **-0.26^<0.0001^** | **-0.27^<0.0001^** | **-0.16^0.009^** | **-0.28^<0.0001^** | -0.11 | **-0.28^<0.0001^** | -0.03 | **-0.25^<0.0001^** | -0.11 | **-0.17^0.005^** | **-0.22^0.0005^** |
| **HAL** | 0.00 | **0.13^0.03^** | **0.41^<0.0001^** | **0.13^0.04^** | **0.32^<0.0001^** | 0.05 | **0.20^0.0008^** | 0.00 | -0.04 | -0.08 | -0.03 |
| **CWN** | 0.06 | 0.03 | 0.03 | 0.03 | 0.01 | 0.00 | 0.01 | 0.02 | -0.08 | 0.09 | 0.10 |
| **CRN** | 0.04 | -0.01 | -0.04 | 0.00 | -0.05 | -0.01 | -0.04 | 0.02 | -0.06 | 0.12 | **0.13^0.046^** |
| **CWC** | **0.32^<0.0001^** | **0.30^<0.0001^** | **0.19^0.002^** | **0.31^<0.0001^** | **0.16^0.006^** | **0.23^<0.0001^** | 0.02 | **0.22^0.0002^** | 0.09 | **0.21^0.0004^** | **0.19^0.003^** |
| **CRC** | **0.28^<0.0001^** | **0.25^<0.0001^** | 0.08 | **0.27^<0.0001^** | 0.07 | **0.21^0.0003^** | -0.04 | **0.20^0.0008^** | 0.11 | **0.22^0.0002^** | **0.20^0.002^** |
| **CWS** | **0.33^<0.0001^** | **0.27^<0.0001^** | **0.26^<0.0001^** | **0.30^<0.0001^** | **0.24^<0.0001^** | **0.26^<0.0001^** | 0.10 | **0.29^<0.0001^** | 0.00 | **0.16^0.01^** | **0.13^0.04^** |
| **CRS** | **0.36^<0.0001^** | **0.27^<0.0001^** | **0.16^0.009^** | **0.30^<0.0001^** | **0.15^0.01^** | **0.28^<0.0001^** | 0.05 | **0.27^<0.0001^** | 0.01 | **0.21^0.0005^** | **0.17^0.008^** |

Abbreviations: VAT, visceral adipose tissue; HOMA-IR, homeostasis model assessment insulin resistance; BTM, bone turnover markers; CTX, C-terminal telopeptide of type 1 collagen; OC, osteocalcin; P1NP, procollagen type 1 N propeptide; BMD, bone mineral density; LS, lumbar spine; FN, femoral neck; TH, total hip; AHA, Advanced hip analysis; BR, buckling ratio; SM, section modulus; CSA, cross sectional area; CSMI, cross sectional moment of inertia; SI, strength index; HAL, hip axis length; CWN, cortical width neck; CRN, cortical ratio neck; CWC, cortical width calcar; CRC, cortical ratio calcar; CWS, cortical width shaft; CRS, cortical ratio shaft.

P-values not listed if >0.05.

|  | **BMI** | **Total Fat** | **Total Lean** | **Trunk Fat** | **Trunk Lean** | **Central Fat** | **Central Lean** | **VAT** | **Glucose** | **Insulin** | **HOMA-IR** |
| --- | --- | --- | --- | --- | --- | --- | --- | --- | --- | --- | --- |
| **BTM** |  |  |  |  |  |  |  |  |  |  |  |
| **CTX** | **-0.21^0.004^** | **-0.14^0.04^** | -0.12 | **-0.19^0.007^** | -0.13 | **-0.15^0.03^** | 0.01 | **-0.20^0.004^** | -0.09 | **-0.37^<0.0001^** | **-0.31^<0.0001^** |
| **OC** | -0.14^NS^ | **-0.17^0.02^** | 0.02 | **-0.21^0.003^** | 0.00 | **-0.20^0.005^** | -0.03 | **-0.19^0.007^** | -0.11 | -0.05 | 0.02 |
| **P1NP** | -0.04^NS^ | -0.06 | 0.09 | -0.10 | 0.09 | -0.13 | 0.06 | -0.10 | **-0.14^0.04^** | -0.02 | -0.01 |
| **BMD** |  |  |  |  |  |  |  |  |  |  |  |
| **LS** | **0.28^<0.0001^** | **0.27^0.0001^** | **0.21^0.03^** | **0.30^<0.0001^** | **0.15^0.04^** | **0.21^0.003^** | -0.04 | **0.22^0.002^** | **0.24^0.0005^** | 0.05 | 0.00 |
| **FN** | **0.29^<0.0001^** | **0.24^0.0005^** | **0.32^<0.0001^** | **0.27^<0.0001^** | **0.28^<0.0001^** | 0.12 | 0.01 | **0.15^0.04^** | -0.04 | -0.04 | -0.04 |
| **TH** | **0.32^<0.0001^** | **0.24^0.0007^** | **0.30^<0.0001^** | **0.27^<0.0001^** | **0.27^0.0001^** | 0.12 | 0.02 | **0.16^0.02^** | 0.02 | 0.06 | 0.05 |
| **AHA** |  |  |  |  |  |  |  |  |  |  |  |
| **BR** | **-0.19^0.01^** | -0.13 | 0.10 | -0.11 | 0.07 | -0.09 | 0.02 | -0.01 | 0.02 | 0.01 | 0.04 |
| **SM** | **0.28^0.0002^** | **0.23^0.002^** | **0.50^<0.0001^** | **0.25^0.0008^** | **0.45^<0.0001^** | 0.08 | 0.12 | **0.16^0.04^** | -0.05 | 0.08 | 0.06 |
| **CSA** | **0.32^<0.0001^** | **0.27^0.0004^** | **0.47^<0.0001^** | **0.30^<0.0001^** | **0.41^<0.0001^** | 0.11 | 0.11 | 0.15 | -0.03 | 0.08 | 0.08 |
| **CSMI** | **0.25^0.001^** | **0.23^0.002^** | **0.52^<0.0001^** | **0.24^0.001^** | **0.46^<0.0001^** | 0.06 | **0.15^0.04^** | 0.14 | -0.05 | 0.07 | 0.05 |
| **SI** | -0.13^NS^ | -0.12 | -0.15 | -0.08 | -0.13 | -0.10 | -0.12 | 0.00 | -0.02 | -0.05 | -0.05 |
| **HAL** | -0.02^NS^ | -0.04 | **0.53^<0.0001^** | -0.07 | **0.47^<0.0001^** | **-0.20^0.01^** | **0.35^<0.0001^** | **-0.21^0.008^** | 0.01 | -0.15 | -**0.20^0.02^** |
| **CWN** | **0.20^0.008^** | **0.16^0.04^** | -0.03 | 0.14 | -0.01 | 0.09 | 0.01 | 0.02 | -0.02 | 0.00 | -0.04 |
| **CRN** | **0.18^0.02^** | 0.13 | -0.11 | 0.11 | -0.08 | 0.09 | -0.01 | 0.01 | -0.02 | -0.02 | -0.05 |
| **CWC** | **0.17^0.02^** | **0.17^0.03^** | 0.07 | **0.22^0.004^** | 0.11 | **0.16^0.04^** | -0.04 | **0.20^0.01^** | **0.19^0.02^** | **0.23^0.003^** | **0.22^0.01^** |
| **CRC** | 0.12^NS^ | 0.13 | -0.04 | **0.18^0.02^** | 0.00 | 0.15 | -0.09 | **0.17^0.03^** | **0.17^0.03^** | **0.21^0.006^** | **0.21^0.01^** |
| **CWS** | 0.13^NS^ | 0.04 | 0.14 | 0.05 | 0.11 | 0.00 | 0.07 | 0.02 | 0.04 | -0.01 | -0.01 |
| **CRS** | 0.08^NS^ | 0.01 | -0.01 | 0.02 | -0.03 | 0.01 | 0.00 | 0.02 | 0.04 | -0.03 | 0.00 |

Abbreviations: VAT, visceral adipose tissue; HOMA-IR, homeostasis model assessment insulin resistance; BTM, bone turnover markers; CTX, C-terminal telopeptide of type 1 collagen; OC, osteocalcin; P1NP, procollagen type 1 N propeptide; BMD, bone mineral density; LS, lumbar spine; FN, femoral neck; TH, total hip; AHA, Advanced hip analysis; BR, buckling ratio; SM, section modulus; CSA, cross sectional area; CSMI, cross sectional moment of inertia; SI, strength index; HAL, hip axis length; CWN, cortical width neck; CRN, cortical ratio neck; CWC, cortical width calcar; CRC, cortical ratio calcar; CWS, cortical width shaft; CRS, cortical ratio shaft.

P-values not listed if >0.05.

## Supplementary Table 2. Linear regression models to explain the variability in BMD, BTM, and AHA according to Principal Components Analysis in women (A) and men (B).

| **Outcome** | **Adjusted R^2^** | **Variable** | **Estimate (95% CI)*** | **P value** |
| --- | --- | --- | --- | --- |
| Bone turnover markers | | | | |
| CTX | 0.052 | PC3 | 6.6% (0.8, 12.7) | 0.03 |
|  |  | PC4 | 10.0% (3.3, 17.2) | 0.003 |
|  |  | PC5 | -6.6% (-12.6, -0.3) | 0.04 |
| OC | 0.088 | PC1 | -2.1% (-3.7, -0.4) | 0.02 |
|  |  | PC4 | 8.3% (4.1, 12.7) | <0.0001 |
|  |  | PC5 | -6.7% (-10.5, -2.7) | <0.001 |
| P1NP | 0.041 | PC5 | -7.6% (-12.1, -2.9) | 0.002 |
| Bone mineral density | | | | |
| FNBMD | 0.144 | PC1 | 0.02 (0.01, 0.02) | <0.0001 |
|  |  | PC3 | -0.03 (-0.04, -0.02) | <0.0001 |
| THBMD | 0.208 | PC1 | 0.02 (0.02, 0.03) | <0.0001 |
|  |  | PC3 | -0.03 (-0.04, -0.02) | <0.0001 |
| LSBMD | 0.098 | PC1 | 0.02 (0.01, 0.03) | <0.0001 |
|  |  | PC3 | -0.02 (-0.04, -0.0007) | 0.04 |
|  |  | PC4 | 0.02 (0.003, 0.04) | 0.03 |
| Advanced hip analysis | | | | |
| BR |  | None |  |  |
| SM | 0.165 | PC1 | 14.25 (9.12, 19.37) | <0.0001 |
|  |  | PC2 | 20.10 (10.87, 29.33) | <0.0001 |
|  |  | PC4 | 16.93 (5.20, 28.66) | 0.005 |
| CSA | 0.193 | PC1 | 2.97 (2.05, 3.90) | <0.0001 |
|  |  | PC2 | 2.33 (0.67, 4.00) | 0.006 |
|  |  | PC3 | -4.14 (-6.02, -2.26) | <0.0001 |
|  |  | PC4 | 2.18 (0.07, 4.30) | 0.04 |
| CSMI | 0.195 | PC1 | 308.73 (205.13, 412.33) | <0.0001 |
|  |  | PC2 | 476.47 (289.88, 663.05) | <0.0001 |
|  |  | PC4 | 388.45 (151.29, 625.62) | 0.001 |
|  |  | PC5 | -263.09 (-510.62, -15.56) | 0.04 |
| SI | 0.078 | PC1 | -0.04 (-0.06, -0.03) | <0.0001 |
| HAL | 0.285 | PC1 | 0.53 (0.30, 0.77) | <0.0001 |
|  |  | PC2 | 1.61 (1.20, 2.02) | <0.0001 |
|  |  | PC4 | 1.47 (0.95, 1.99) | <0.0001 |
| CWN |  | None |  |  |
| CRN |  | None |  |  |
| CWC | 0.091 | PC1 | 3.3% (2.0, 4.6) | <0.0001 |
|  |  | PC3 | -2.9% (-5.4, -0.3) | 0.03 |
| CRC | 0.085 | PC1 | 2.5% (1.2, 3.8) | 0.0002 |
|  |  | PC2 | -3.5% (-5.7, -1.2) | 0.004 |
|  |  | PC3 | -3.3% (-5.8, -0.7) | 0.01 |
| CWS | 0.096 | PC1 | 3.9% (2.5, 5.4) | <0.0001 |
| CRS | 0.091 | PC1 | 3.1% (1.6, 4.5) | <0.0001 |
|  |  | PC2 | -2.7% (-5.1, -0.2) | 0.03 |

A. Analyses in the whole cohort of women.

PC1 = body size, PC2 = lean mass, PC3 = age, PC4 = mixed, PC5 = lifestyle.

*For the variables that were log-transformed, parameter estimates have been back-transformed, to denote the % change in the dependent variable with every 1% change in the independent variable. Non log-transformed variables in displayed units. Only the PC that were significant are listed.

| **Outcome** | **Adjusted R^2^** | **Variable** | **Estimate (95% CI)** | **P value** |
| --- | --- | --- | --- | --- |
| Bone turnover markers | | | | |
| CTX | 0.022 | PC1 | -3.9% (-6.6, -1.1) | 0.006 |
| OC | 0.029 | PC1 | -2.0% (-3.9, -0.1) | 0.04 |
| P1NP | 0.016 | PC2 | 3.8% (0.6, 7.1) | 0.02 |
| Bone mineral density | | | | |
| FNBMD | 0.092 | PC1 | 0.02 (0.008, 0.02) | <0.0001 |
| THBMD | 0.069 | PC1 | 0.02 (0.009, 0.03) | 0.0001 |
| LSBMD | 0.109 | PC1 | 0.03 (0.01, 0.04) | <0.0001 |
|  |  | PC3 | 0.03 (0.01, 0.06) | 0.005 |
| Advanced hip analysis | | | | |
| BR | 0.043 | PC2 | 4.2% (0.5, 8.1) | 0.03 |
|  |  | PC5 | -7.1% (-12.5, -1.2) | 0.08 |
| SM | 0.258 | PC1 | 33.89 (22.72, 45.06) | <0.0001 |
|  |  | PC2 | 32.18 (17.42, 46.93) | <0.0001 |
|  |  | PC5 | -35.04 (-59.44, -10.65) | 0.005 |
| CSA | 0.220 | PC1 | 4.72 (3.11, 6.32) | <0.0001 |
|  |  | PC2 | 3.91 (1.79, 6.03) | 0.0004 |
|  |  | PC5 | -4.46 (-7.97, -0.95) | 0.01 |
| CSMI | 0.292 | PC1 | 814.37 (554.69, 1074.04) | <0.0001 |
|  |  | PC2 | 876.84 (533.52, 1219.55) | <0.0001 |
|  |  | PC3 | 496.47 (22.10, 970.84) | 0.04 |
|  |  | PC5 | -846.13 (-1413.06, -279.21) | 0.004 |
| SI |  | None |  |  |
| HAL | 0.405 | PC1 | 0.52 (0.13, 0.92) | 0.01 |
|  |  | PC2 | 2.46 (1.95, 2.98) | <0.0001 |
|  |  | PC5 | -1.63 (-2.51, -0.75) | 0.0003 |
| CWN | 0.031 | PC5 | 6.3% (0.2, 12.7) | 0.04 |
| CRN | 0.047 | PC2 | -4.0% (-7.4, -0.5) | 0.03 |
|  |  | PC5 | 7.8% (1.6, 14.3) | 0.01 |
| CWC | 0.021 | PC1 | 2.8% (0.3, 5.3) | 0.03 |
| CRC |  | None |  |  |
| CWS | 0.056 | PC4 | -7.8% (-12.1, -3.2) | 0.001 |
| CRS | 0.015 | PC4 | -6.8% (-11.5, -1.8) | 0.009 |

B. Analyses in the whole cohort of men.

PC1 = body size, PC2 = lean mass, PC3 = age, PC4 = alcohol, PC5 = smoking.

*For the variables that were log-transformed, parameter estimates have been back-transformed, to denote the % change in the dependent variable with every 1% change in the independent variable. Non log-transformed variables in displayed units. Only the PC that were significant are listed.
